# Group 3 Innate Lymphoid Cells Protect the Host from the Uropathogenic *Escherichia coli* Infection in the Bladder

**DOI:** 10.1002/advs.202103303

**Published:** 2022-01-12

**Authors:** Jiaoyan Huang, Liuhui Fu, Jida Huang, Jie Zhao, Xin Zhang, Wenyan Wang, Yeyang Liu, Bowen Sun, Ju Qiu, Xiaoyu Hu, Zhihua Liu, Xiaohuan Guo

**Affiliations:** ^1^ Institute for Immunology Tsinghua University Beijing 100084 China; ^2^ Department of Basic Medical Sciences School of Medicine Tsinghua University Beijing 100084 China; ^3^ Beijing Key Lab for Immunological Research on Chronic Diseases Tsinghua University Beijing 100084 China; ^4^ CAS Key Laboratory of Tissue Microenvironment and Tumor Shanghai Institute of Nutrition and Health Chinese Academy of Sciences Shanghai 200031 China

**Keywords:** bladder, IL‐17A, IL‐1*β*, ILC3, innate lymphoid cells, uropathogenic *Escherichia coli* infection

## Abstract

Innate lymphoid cells (ILCs) are crucial in orchestrating immunity and maintaining tissue homeostasis in various barrier tissues, but whether ILCs influence immune responses in the urinary tract remains poorly understood. Here, bladder‐resident ILCs are comprehensively explored and identified their unique phenotypic and developmental characteristics. Notably, bladder‐resident ILCs rapidly respond to uropathogenic *Escherichia coli* (UPEC) infection. It is found that ILC3 is necessary for early protection against UPEC infection in the bladder. Mechanistically, UPEC infection leads to interleukin (IL)‐1*β* production in the bladder via a MyD88‐dependent pathway, which promotes ILC3 activation. ILC3‐expressed IL‐17A further recruits neutrophils and controls UPEC infection in the bladder. Together, these results demonstrate a critical role for bladder ILCs in the host defense against UPEC infection.

## Graphical abstract

Urinary tract infections by uropathogenic *Escherichia coli* (UPEC) are one of the most common bacterial infections worldwide. Innate lymphoid cells (ILCs), a novel population of immune cells, are found to be resident in the urinary tract. UPEC infection drives a rapid ILC3 response in the bladder. ILC3s further protect the host from UPEC infection through interleukin‐17‐mediated neutrophil recruitment.

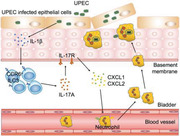

## 1 Introduction

Innate lymphoid cells (ILCs) represent a novel population of innate immune cells that exhibit functional characteristics similar to those of T cells, but lack the expression of rearranged antigen receptors.^[^ ^1^ ^]^ Based on the expression profiles of transcription factors and effector cytokines, ILCs can be divided into three subsets: group 1 ILCs, including natural killer (NK) cells and ILC1; group 2 ILCs (ILC2); and group 3 ILCs (ILC3). Although both NK cells and ILC1 express the transcription factor T‐bet, ILC1 mainly produces interferon *γ* (IFN‐*γ*) and tumor necrosis factor *α* (TNF‐*α*) and is less cytotoxic compared with conventional NK cells. ILC2 expresses the transcription factor GATA binding protein 3 (GATA3) and secretes type 2 cytokines such as interleukin (IL)‐5 and IL‐13 in response to IL‐25 or IL‐33. As the innate counterpart of T helper (Th)17 and Th22 cells, the transcription factor retinoic acid receptor‐related orphan nuclear receptor gamma t (ROR*γ*t)‐expressing ILC3 is a heterogeneous population, including chemokine receptor 6 (CCR6)^+^ lymphoid tissue inducer (LTi) or LTi‐like cells (an adult version of LTi), natural cytotoxicity receptor (NCR)^+^ and NCR^−^ ILC3s. LTi and LTi‐like cells can express high levels of lymphotoxin and are important for the formation of secondary or tertiary lymphoid structures, whereas NCR^+^ and NCR^−^ ILC3s are considered the major innate producers of IL‐22 in response to IL‐23 or IL‐1*β* stimulation.^[^ ^2^ ^]^

Multiple factors are involved in the regulation of ILC development and function. These include not only cell‐intrinsic transcription factors, such as inhibitor of DNA binding 2 (ID2), promyelocytic leukemia zinc finger (PLZF), GATA3, ROR*γ*t, and signal transducer and activator of transcription 3 (STAT3),^[^ ^3^, ^4^, ^5^, ^6^, ^7^ ^]^ but also environmental factors, such as common *γ*‐chain family cytokines (IL‐2, IL‐7, and IL‐15), Notch, and even microbiota signaling.^[^ ^8^, ^9^, ^10^ ^]^ ILCs primarily reside in barrier tissues, such as the intestine, lungs, and skin, and respond quickly to various stresses and pathogen invasion. Thus, ILCs play key roles in not only tissue homeostasis, but also in many diseases, such as infection, inflammation, autoimmunity, and tumors.^[^ ^11^, ^12^, ^13^ ^]^ Additionally, it has previously been reported that ILCs can be imprinted by different tissue signals, and even the same subset of ILCs exhibit heterogeneity between different tissues.^[^ ^14^, ^15^, ^16^ ^]^ The urinary tract is one of the most important barrier tissues in the body, and comprises the kidneys, ureters, bladder, and urethra.^[^ ^17^ ^]^ Several studies have shown that ILC2s represent a major ILC population in the murine kidney and protect the host from both acute kidney injury and chronic kidney disease.^[^ ^18^, ^19^, ^20^ ^]^ Several groups have shown the role of ILC2 during bladder cancer recurrence^[^ ^21^, ^22^ ^]^ and the protection role of NK cells in uropathogenic *Escherichia coli* (UPEC) infection.^[^ ^23^, ^24^ ^]^ However, a comprehensive examination of the characteristics of bladder‐resident ILCs is lacking, and whether these bladder‐resident ILCs share similar characteristics with ILCs in other barrier tissues is still unknown.

Urinary tract infections (UTIs) are one of the most common bacterial infections worldwide and exhibit significantly greater incidence in females than males. The major cause of UTIs is UPEC, which is usually found in the gastrointestinal tract but can infect the urinary tract, particularly the bladder and urethra.^[^ ^25^, ^26^ ^]^ UPEC induces both innate and adaptive immune responses in the urinary tract.^[^ ^27^, ^28^, ^29^, ^30^ ^]^ However, due to ineffective adaptive memory immunity, many patients with UPEC develop recurrent infections. This can lead to serious antibiotic resistance,^[^ ^26^ ^]^ and there is thus an urgent need for in‐depth understanding of immunity against UTIs. In the bladder and urethra, stratified epithelial cells are the first line of protection against UPEC infection. Under the basal epithelium, the resident mast cells, NK cells, Ly6C^−^ macrophages, and *γδ*T cells function as sentinels to sense UPEC infection, and neutrophils and Ly6C^+^ macrophages are further recruited into the bladder to eliminate the bacteria.^[^ ^17^, ^23^, ^31^, ^32^, ^33^, ^34^, ^35^ ^]^ ILCs play a critical role in the host defense against pathogens in other barrier tissues, such as the gut, lung, and skin,^[^ ^12^, ^13^ ^]^ and it has been reported that CD4^+^ ILC3 levels are increased in the bladder of UPEC‐infected mice.^[^ ^36^ ^]^ However, whether bladder‐resident ILCs respond to UPEC infection, and whether ILC3s play a crucial role in defending against UPEC during an infection, remain to be determined.

Here, we comprehensively explored the phenotypic and developmental characteristics of ILCs in the bladder, and found that ILC3s are essential for protection against UPEC through regulation of neutrophil recruitment.

## 2 Results

### 2.1 Bladder‐Resident ILCs Exhibit Distinct Characteristics Compared with ILCs in the Gut

**Figure 1:**
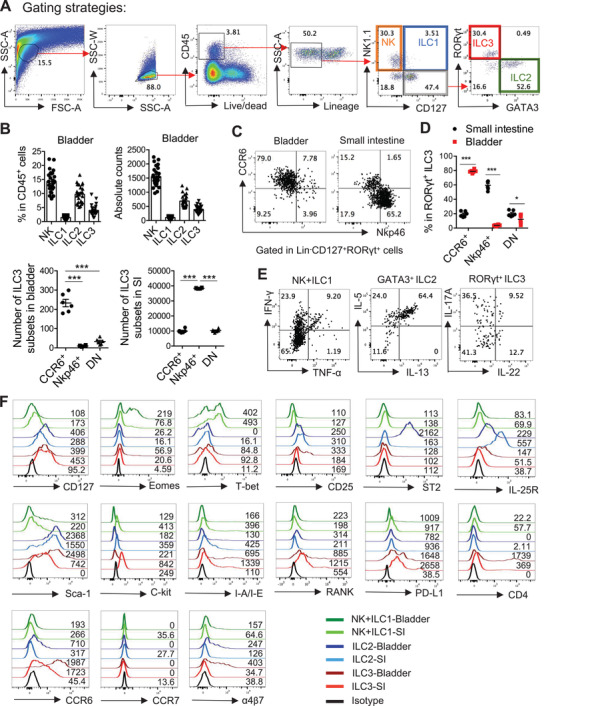
Bladder‐resident ILCs exhibit distinct characteristics. A) Gating strategy used to identify the ILC subsets. The whole bladder tissue was digested and further analyzed by flow cytometry. NK cells were defined as live CD45^+^Lin^−^NK1.1^+^CD127^−^ cells; ILC1 were defined as live CD45^+^Lin^−^NK1.1^+^CD127^+^ cells; ILC2 were defined as live CD45^+^Lin^−^NK1.1^−^CD127^+^GATA3^+^ cells, and ILC3 were defined as live CD45^+^Lin^−^NK1.1^−^CD127^+^ROR*γ*t^+^ cells. (Lin, Lineage markers including CD8, CD3ɛ, TCR*β*, TCR*γδ*, B220, Ter119, and GR‐1). The red arrows show the gating steps. B) The percentages in CD45^+^ cells and the absolute numbers of different ILC subsets from the bladder of WT mice are shown (n = 25 mice). Data are pooled from five independent experiments. C) Representative plots show CCR6 and NKp46 expression on the ROR*γ*t^+^ ILC3 in the bladder and the small intestine of WT mice. D) The percentages in total ROR*γ*t^+^ ILC3 and the absolute numbers of ILC3 subsets (CCR6^+^, NKp46^+^, and CCR6^−^NKp46^−^ cells) from the bladder and the small intestine of WT mice are shown (n = 6 mice). Data are pooled from two independent experiments. E) IFN‐*γ* and TNF‐*α* expression in NK1.1^+^ ILCs (NK cells and ILC1), IL‐13 and IL‐5 expression in GATA3^+^ ILC2 and IL‐22 and IL‐17A expression in ROR*γ*t^+^ ILC3 were analyzed by intracellular cytokine staining followed by flow cytometry. The bladder tissues of naïve WT mice were digested and cells were stimulated with PMA (50 ng mL^−1^) and ionomycin (750 ng mL^−1^) for 4 h and further gated in CD45^+^CD3ɛ^−^ cells. F) Histograms show the expression level of indicated cell surface markers, transcription factors, and chemokine receptors on ILC subsets in the bladder and the small intestine. The value of mean fluorescent intensity has been labeled. Data are shown as the mean ± SEM. Each dot represents one individual mouse. Two‐way ANOVA followed by Bonferroni test was used for statistical analysis, *p<0.05, ***p<0.001. Data shown are representative of two or three independent experiments (A, C, E, F).

To comprehensively identify the distribution of ILC subsets in the urinary tract, leukocytes were isolated from the bladder and kidney of naïve wild type (WT) mice and analyzed by flow cytometry. Based on the expression of ILC‐specific surface markers and transcription factors, all three ILC groups were detected in the bladder and kidney (**Figure** **1**A,B and Figure S1A, Supporting Information). Among ILCs, NK cells were the dominant population in both the bladder and kidney, but only a few ILC1s could be detected. Consistent with previous reports,^[^ ^20^, ^21^ ^]^ ILC2s were found in both tissues, whereas ILC3s were mainly resident in the bladder (Figure 1B and Figure S1A, Supporting Information). Interestingly, unlike intestinal ILC3s, most ILC3s in the urinary tract were CCR6^+^NKp46^−^ LTi‐like cells (Figure 1C,D and Figure S1B,C, Supporting Information). As previously reported,^[^ ^2^, ^37^, ^38^ ^]^ these ILC subsets from the bladder also produced corresponding effector cytokines post‐stimulation: group 1 ILCs (NK and ILC1) produced IFN‐*γ* and TNF‐*α*; ILC2s produced IL‐5 and IL‐13; and ILC3s produced IL‐17A and IL‐22 (Figure 1E). Because ILCs are known to display heterogeneity between different tissues,^[^ ^15^, ^16^, ^39^ ^]^ ILC surface markers and transcription factors were examined to further characterize the bladder ILCs (Figure 1F). Compared with ILCs from the small intestine, bladder‐resident group 1 ILCs showed increased expression of the Eomes, which may reflect the presence of more NK cells in the bladder than in the small intestine; bladder‐resident ILC2s exhibited higher IL‐33 receptor ST2 but lower IL‐25 receptor (IL‐25R) expression, suggesting that bladder ILC2s may respond well to IL‐33, but not to IL‐25, at the naïve stage. Bladder‐resident ILC3s highly expressed CCR6; however, compared with intestinal ILC3s, bladder‐resident ILC3s had reduced expression of major histocompatibility complex class II (MHC‐II), programmed death‐ligand 1 (PD‐L1), and receptor activator of nuclear factor‐*κ*B (RANK), which are important for the interaction between ILC3 and T cells.^[^ ^16^ ^]^ This suggests that ILC3s in the bladder may not regulate T cell immunity. Additionally, bladder ILC3s also expressed less tyrosine‐protein kinase Kit (c‐Kit) but more stem cell antigen‐1 (Sca‐1), suggesting that different regulation mechanisms may be involved in ILC3 proliferation in different tissues. Moreover, although most bladder‐resident ILC3s were CCR6^+^ LTi‐like cells, only a small proportion expressed CD4, consistent with a previous report that detected only small amounts of CD4^+^ ILC3s in the bladder.^[^ ^36^ ^]^ Notably, compared with intestinal ILCs, both ILC2s and ILC3s expressed higher integrin *α*4*β*7 and chemokine receptor CCR6 levels in the bladder, indicating that the migration of ILC2s and ILC3s into the bladder may be dependent on these homing molecules (Figure 1F). Together, these data suggest that ILCs residing in the bladder display unique characteristics.

### 2.2 Common *γ*‐Chain Family Cytokines and ID2 but not Commensal Microbes are Required for the Development of Bladder‐Resident ILCs

**Figure 2:**
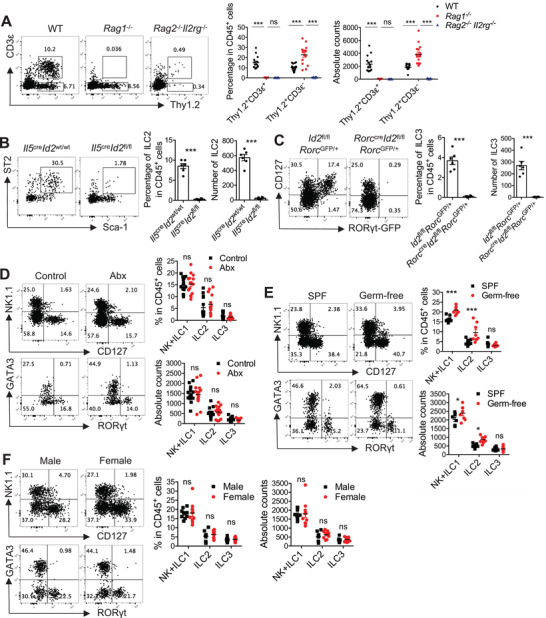
Common *γ*‐chain family cytokines and ID2 but not commensal are required for the maintenance of bladder‐resident ILCs. A) Representative plots, percentages, and absolute numbers of the gated T cells (Thy1.2^+^CD3ɛ^+^ cells) and ILCs (Thy1.2^+^CD3ɛ^−^ cells) in the bladder of WT, *Rag1* ^−/‐^ and *Rag2* ^−/−^ *Il2rg* ^−/‐^ mice are shown (n = 15 mice). Data are pooled from three independent experiments. B) Representative plots, percentages and absolute numbers of the gated ILC2 (Lin^−^CD127^+^ST2^+^Sca‐1^+^ cells) in the bladder of *Il5* ^tdtomato‐cre^ *Id2* ^wt/wt^ and *Il5* ^tdtomato‐cre^ *Id2* ^fl/fl^ mice are shown (n = 6 mice). Data are pooled from two independent experiments. C) Representative plots, percentages and absolute numbers of the gated ILC3 (Lin^−^CD127^+^ROR*γ*t‐GFP^+^ cells) in the bladder of *Id2* ^fl/fl^ *Rorc* ^GFP/+^ and *Rorc* ^cre^ *Id2* ^fl/fl^ *Rorc* ^GFP/+^ mice are shown (n = 6 mice). Data are pooled from two independent experiments. D) Representative plots, percentages, and absolute numbers of different ILC subsets in the bladder of the antibiotics‐treated (Abx) mice and the control C57BL/6 mice are shown (n = 14–15 mice). Data are pooled from three independent experiments. E) Representative plots, percentages, and absolute numbers of different ILC subsets in the bladder of germ‐free and SPF C57BL/6 mice are shown (n = 8 mice). Data are pooled from two independent experiments. F) Representative plots, percentages, and absolute numbers of different ILC subsets in the bladder of male and female C57BL/6 mice are shown (n = 10 mice). Data are pooled from two independent experiments. Mice in these experiments are adults. Data are shown as mean ± SEM. Two‐way ANOVA followed by Bonferroni test (A, D–F) and unpaired two‐tails student's *t*‐test (B, C) were conducted for statistical analysis, *p<0.05, ***p<0.001; ns, no significant difference.

The common *γ*‐chain family cytokines comprise IL‐2, IL‐4, IL‐7, IL‐9, IL‐15, and IL‐21, and share the common cytokine receptor *γ* chain (*γ*c) encoded by *Il2rg*. Previous studies have shown that IL‐2, IL‐7, and IL‐15 are required for the development of ILCs in various tissues.^[^ ^40^ ^]^ Consistently, ILC levels were dramatically reduced in *Rag2* ^−/^ *^−^Il2rg* ^−/−^ mice compared with WT or *Rag1* ^−/−^ mice (**Figure** **2**A), suggesting that common *γ*‐chain family cytokines are also required for the development of bladder‐resident ILCs. ID2 is an essential transcriptional regulator for the development of all ILCs.^[^ ^1^ ^]^ We previously found that ID2 differentially regulated ILC3s in the mesenteric lymph node (mLN) and gut.^[^ ^3^, ^16^ ^]^ To determine the role of ID2 in the development of bladder‐resident ILCs, *Id2* ^fl/fl^ mice were crossed with *Il5* ^tdtomato‐cre^ and with *Rorc* ^cre^ mice to specifically delete ID2 in ILC2s or ILC3s. Results showed that ID2 deficiency severely depleted ILC2s and ILC3s (Figure 2B,C), indicating that ID2 is essential for the maintenance of ILC2s and ILC3s in the bladder.

Commensal microbiota can modulate the host immune system, including ILCs.^[^ ^9^, ^41^ ^]^ To determine whether microbiota‐derived signals regulate the development of bladder‐resident ILCs, WT mice were treated with an antibiotic cocktail to clear the gut of microbiota.^[^ ^42^ ^]^ Antibiotic treatment did not exhibit significant effects on ILCs in the bladder of naïve mice (Figure 2D). We further examined the bladder‐resident ILCs from conventional specific‐pathogen‐free (SPF) and germ‐free mice. Interestingly, there was no difference in ILC3 levels, but a slight increase in ILC1 and ILC2 levels in the bladder of germ‐free mice compared with SPF mice (Figure 2E). Additionally, both antibiotic‐treated and germ‐free mice showed few differences in the proportions and absolute numbers of ILCs in the kidney (Figure S2A,B, Supporting Information). These data indicate that the development of ILCs in the urinary tract may not rely on signals derived from commensal bacteria.

Female individuals generally have a much higher incidence of UTIs than males,^[^ ^43^ ^]^ and it has been reported that sex influences the immune responses during chronic UTI.^[^ ^36^ ^]^ We therefore compared ILC levels in the bladder and kidney of male and female mice. There were no significant differences in ILC levels between the male and female urinary tract (Figure 2F and Figure S2C, Supporting Information), suggesting that sex is not critical for the maintenance of ILCs in the urinary tract.

Collectively, these data demonstrate that the maintenance of bladder‐resident ILCs requires common *γ*‐chain family cytokines and ID2, but not commensal‐ or sex‐related signals at the naïve stage.

### 2.3 UPEC Infection Drives a Rapid ILC Immune Response in the Bladder

**Figure 3:**
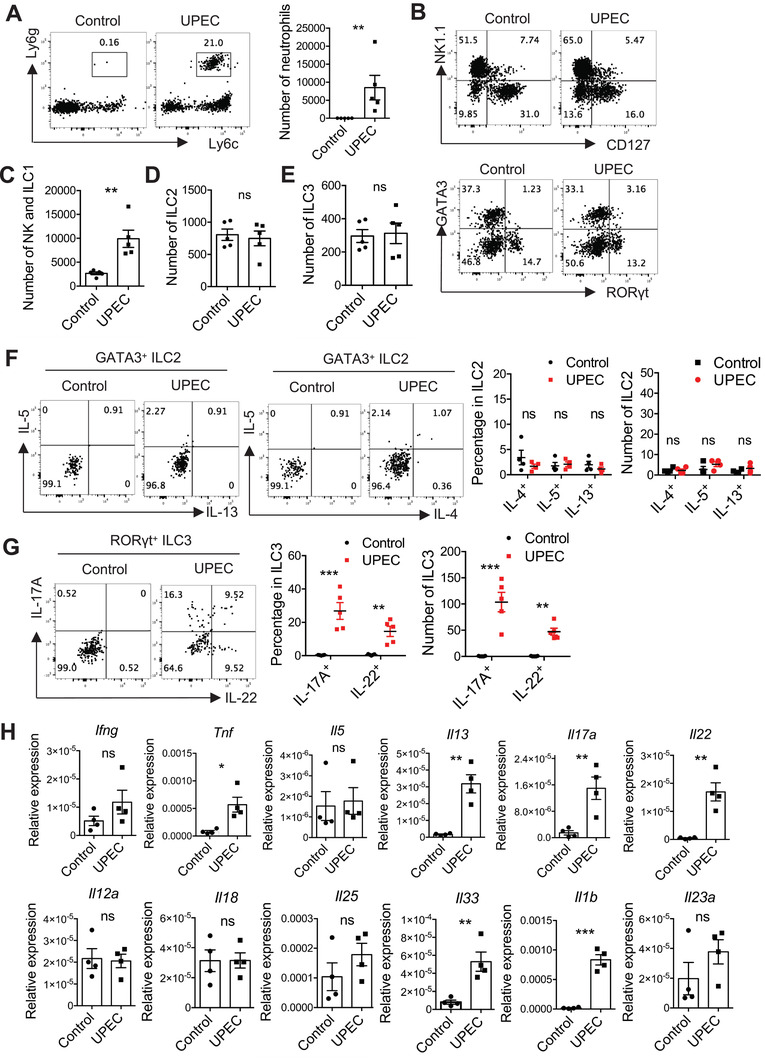
UPEC infection drives a rapid innate immune response in the bladder. 8–10 weeks‐old WT female mice were transurethrally infected with 1×10^8^ CFU of UPEC or PBS control. The bladder tissues were isolated at 18h post‐treatment. A) Representative plots and the absolute number of neutrophils (CD45^+^Ly6c^med^Ly6g^+^ cells) in the whole bladder are shown (n = 5 mice). (B–E) B) Representative plots and the absolute number of C) NK1.1^+^ ILCs, D) ILC2, and E) ILC3 in the whole bladder are shown (n = 5 mice). (F, G) The bladder tissue was digested and cells were treated with Brefeldin A (5 µg mL^−1^) for 3 h. F) IL‐4, IL‐5, and IL‐13 expression in GATA3^+^ ILC2, G) IL‐17A and IL‐22 expression in ROR*γ*t^+^ ILC3 were analyzed by intracellular cytokine staining followed by flow cytometry. Representative plots, percentages, and absolute numbers of these cytokines‐producing cells are shown (n = 4–5 mice). H) The mRNA expression of the indicated type 1, type 2, and type 3 cytokines in the bladder tissues were measured by real‐time RT‐PCR (n = 4 mice). Data are representative of three independent experiments. Error bars represent the mean ± SEM. Two‐way ANOVA followed by Bonferroni test (F, G) and unpaired two‐tails student's *t*‐test (A, C–E, and H) were used for statistical analysis, *p<0.05, **p<0.01, ***p<0.001; ns, no significant difference.

ILCs play a critical role in the host defense against pathogens in barrier tissues.^[^ ^44^, ^45^, ^46^, ^47^ ^]^ It has been shown that NK cells secrete TNF‐*α* and restrict UPEC infection in the bladder.^[^ ^23^, ^24^ ^]^ To determine whether other ILCs in the bladder are also involved in defense against UTIs, WT mice were transurethrally infected with UPEC. UPEC infection rapidly induced an accumulation of neutrophils in the bladder (**Figure** **3**A), which is one of the hallmarks of a UTI.^[^ ^48^, ^49^ ^]^ Consistent with previous reports,^[^ ^23^, ^24^ ^]^ NK cell level was significantly increased and TNF‐*α* production in the bladder was induced upon UPEC infection (Figure 3B,C,H). Interestingly, although the number of bladder‐resident ILC3s was not changed (Figure 3B,E), significant activation of ILC3s was observed through increased IL‐17A and IL‐22 production after UPEC challenge (Figure 3G). Consistently, *Il17a*, *Il22*, and the ILC3 upstream stimulator *Il1b* (but not *Il23a*) were markedly up‐regulated in the bladder after UPEC infection (Figure 3H). Notably, although no obvious differences in the number or activation state of ILC2s were detected (Figure 3B,D,F), we also found that the type 2 cytokine *Il13* and the related alarmin *Il33* were significantly up‐regulated after infection (Figure 3H). This suggested that other type 2 immune cells, not ILC2s, may be involved in responding to UTI. Together, these data indicate that UPEC infection drives a rapid immune response by ILCs, and that in addition to NK cells, ILC3s may also contribute to the host defense against UTI.

### 2.4 ILCs are Required for Resistance to UPEC Infection

**Figure 4:**
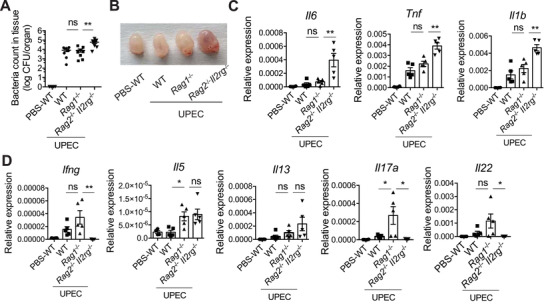
ILCs‐deficient mice are more susceptible to the UTI. 8 weeks‐old WT, *Rag1* ^−/‐^ and *Rag2* ^−/−^ *Il2rg* ^−/‐^ female mice were transurethrally infected with 1×10^8^ CFU of UPEC or PBS. The bladder tissues at 18h post‐treatment were isolated. A) The UPEC burdens in the whole bladder at 18 h post‐infection are shown (n = 7–8 mice). B) The representative image of the bladders for each group is shown. C) The mRNA expression of the indicated pro‐inflammatory cytokines in the bladder is shown (n = 4–5 mice). D) The mRNA expression of the indicated effector cytokines of ILCs in the bladder is shown (n = 4–5 mice). Data are representative of three independent experiments. Error bars represent the mean ± SEM. One‐way ANOVA test (A, C, D) was used for statistical analysis, *p<0.05, **p<0.01; ns, no significant difference.

To identify the role of ILCs in response to UTI, WT, *Rag1* ^−/−^, and *Rag2* ^−/−^ *Il2rg* ^−/^ *^‐^* mice were challenged with UPEC. At 18 h post‐infection, WT mice and *Rag1* ^−/‐^ mice showed comparable UPEC burdens in the bladder, whereas ILCs‐deficient *Rag2* ^−/−^ *Il2rg* ^−/‐^ mice exhibited a significant increase in UPEC burden compared with WT and *Rag1* ^−/‐^ mice (**Figure** **4**A). This suggests an important role of ILCs in the early control of UPEC infection in the bladder. Consistently, the bladders of *Rag2* ^−/^ *^−^Il2rg* ^−/‐^ mice also displayed more severe inflammatory symptoms than WT and *Rag1* ^−/‐^ mice, including hyperemia and edema (Figure 4B) and higher expression of pro‐inflammatory cytokines including *Il6*, *Tnf*, and *Il1b* (Figure 4C). Expression of ILC‐related cytokines was also examined. As expected, *Rag2* ^−/−^ *Il2rg* ^−/‐^ mice did not show up‐regulation of *Ifng*, *Il17a*, or *Il22* (Figure 4D), indicating that NK cells and ILC3s are the major producers of IFN‐*γ*, IL‐17A, and IL‐22. However, ILC deficiency did not affect the expression of *Il5* and *Il13* (Figure 4D), suggesting that ILC2 may not be the major producer of type 2 cytokines in the bladder.

To further confirm the role of ILC2s in UPEC infection, ILC2‐deficient *Il5* ^tdtomato‐cre^ *Id2* ^fl/fl^ and control mice were infected with UPEC. Although ID2 deficiency was associated with a reduction in IL‐5 and IL‐13 expression (Figure S3C, Supporting Information), the UPEC burdens and expression of pro‐inflammatory cytokines in the bladder were not significantly different between ILC2‐deficient *Il5* ^tdtomato‐cre^ *Id2* ^fl/fl^ and control mice (Figure S3A,B, Supporting Information). This suggested that ILC2s are not required for early control of UPEC infection in the bladder. Together, these results suggest that NK cells and ILC3s may play important roles in protecting the host from UPEC infection at the acute stage.

### 2.5 ILC3 Plays an Essential Role in Early Protection from UTI

**Figure 5:**
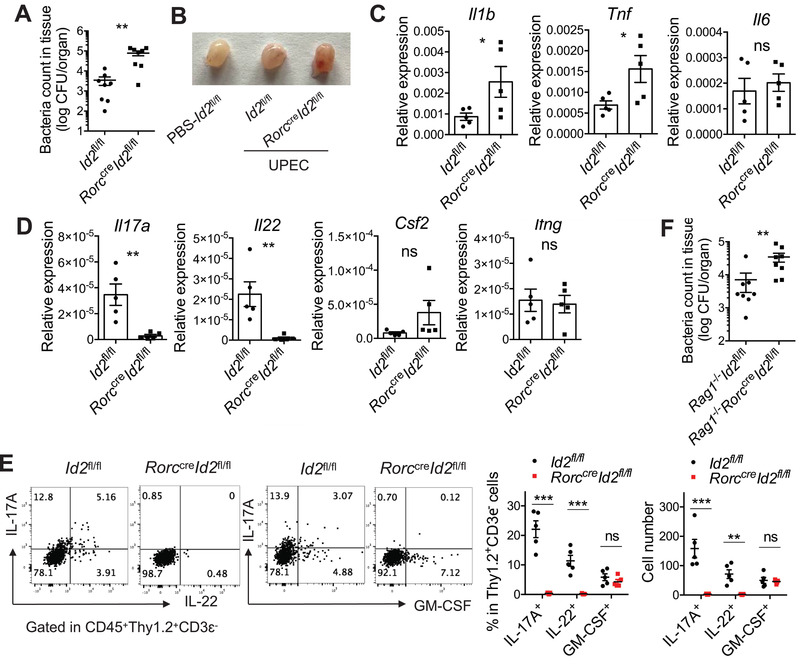
Bladder‐resident ILC3 plays an essential role in the defense against UTI. 8 weeks‐old *Id2* ^fl/fl^ and *Rorc* ^cre^ *Id2* ^fl/fl^ female mice were transurethrally infected with 1×10^8^ CFU of UPEC. The bladder tissues were isolated at 18 h post‐treatment. A) The UPEC burdens in the whole bladder tissue are shown (n = 8 mice). B) The representative image of the bladders for each group is shown. C) The mRNA expression of the indicated pro‐inflammation cytokines in the infected bladder tissues is shown (n = 5 mice). D) The mRNA expression of the indicated effector cytokines of ILC3 in the infected bladder tissues is shown (n = 5 mice). E) IL‐22, IL‐17A, and GM‐CSF expression in the CD45^+^Thy1.2^+^CD3ɛ^−^ cells were analyzed by intracellular cytokine staining. Representative plots, percentages, and absolute numbers of these cytokines‐producing cells are shown. The bladder tissues were digested and cells were treated with Brefeldin A (5 µg mL^−1^) for 3 h (n = 4 mice). Data are representative of three independent experiments. F) 8 weeks‐old *Rag1* ^−/−^ *Id2* ^fl/fl^ and *Rag1* ^−/−^ *Rorc* ^cre^ *Id2* ^fl/fl^ female mice were transurethrally infected with 1×10^8^ CFU of UPEC. The UPEC burdens in the whole bladder tissue at 18 h after infection are shown (n = 8 mice). Data are representative of three independent experiments. Error bars represent the mean ± SEM. Two‐way ANOVA followed by Bonferroni test (E) and unpaired two‐tails student's *t*‐test (A, C–F) were used for statistical analysis, *p<0.05, **p<0.01, ***p<0.001; ns, no significant difference.

NK cell‐mediated host defense against UPEC infection has been reported.^[^ ^23^, ^24^ ^]^ Therefore, we next examined the role of bladder‐resident ILC3s during UPEC infection using *Rorc* ^cre^ *Id2* ^fl/fl^ mice, in which the development of bladder‐resident ILC3s was dramatically disrupted (Figure 2C). Similar to the *Rag2* ^−/−^ *Il2rg* ^−/‐^ mice (Figure 4A–C), *Rorc* ^cre^ *Id2* ^fl/fl^ mice had significantly increased pathogen burdens, inflammation, and pro‐inflammatory cytokine expression in the bladder compared to control mice at early time points (**Figure** **5**A–C). Consistently, the expression of IL‐17A and IL‐22, but not of granulocyte macrophage colony‐stimulating factor (GM‐CSF) and IFN‐*γ*, were reduced in the ILC3‐deficient mice (Figure 5D,E). Because *Rorc* ^cre^ mice can also target T cells,^[^ ^50^ ^]^ we excluded ID2's role in T cells in our model by crossing *Rorc* ^cre^ *Id2* ^fl/fl^ with the T cell‐deficient *Rag1* ^−/−^ mice. Compared with control mice, *Rorc* ^cre^ *Id2* ^fl/fl^ *Rag1* ^−/−^ mice also had decreased ability to eliminate UPEC in the bladder at early time points (Figure 5F). Together, these data demonstrate that ILC3s are essential for early protection from UPEC infection.

Previous studies have shown that IL‐17 and IL‐17‐producing *γδ*T cells play a critical role in protection against UPEC infection.^[^ ^33^ ^]^ However, based on the UPEC burdens in WT and *Rag1* ^−/−^ mice, our results suggest that T cells may not be essential in early defense (Figure 4A). Additionally, expression levels of *Il17a* and *Il22* were much higher in the UPEC‐infected bladder tissues of *Rag1* ^−/−^ mice than WT mice (Figure 4D). We therefore speculated that IL‐17‐ and IL‐22‐producing ILC3s may compensate for the role of *γδ*T cell in *Rag1* ^−/−^ mice. To test this hypothesis, the abundance and functions of bladder ILC3s in *Rag1* ^−/−^ mice and WT mice were further compared. Flow cytometry showed that the absolute numbers of ILC3s (Figure 2A and Figure S4A, Supporting Information) and IL‐17A‐ and IL‐22‐producing ILC3s after UPEC infection (Figure S4B, Supporting Information) were both increased in the bladder of *Rag1* ^−/−^ compared to WT mice. This suggests that ILC3 in the bladder may compensate for T cell deficiency during UTI.

### 2.6 Bladder‐Derived IL‐1*β* Induces ILC3 Activation in Acute UTI

**Figure 6:**
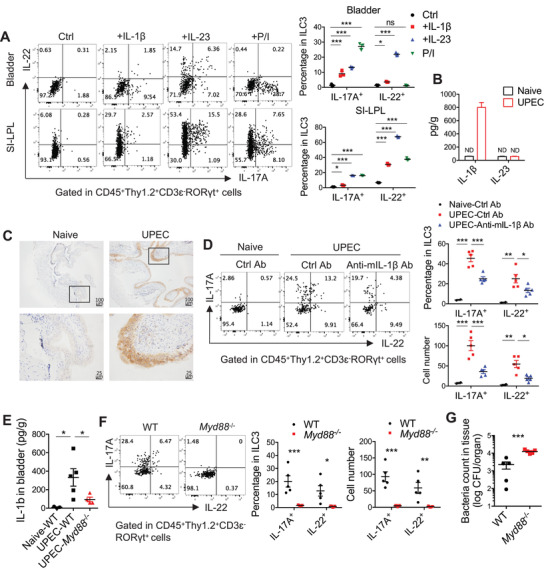
Bladder‐derived IL‐1*β* induces ILC3 activation in the UTI. A) The bladder leukocytes and the leukocytes in small intestinal lamina propria (SI‐LPL) were isolated from naïve WT mice and stimulated with IL‐1*β* (10 ng mL^−1^), IL‐23 (10 ng mL^−1^) or PMA (50 ng mL^−1^), and ionomycin (750 ng mL^−1^) for 4 h. IL‐17A and IL‐22 expression in ROR*γ*t^+^ ILC3 were analyzed by intracellular cytokine staining followed by flow cytometry. Representative plots and the percentages of these cytokines‐producing cells in bladder ILC3 or small intestinal ILC3 are shown (n = 3). (B, C) 8 weeks‐old WT female mice were transurethrally infected with 1×10^8^ CFU of UPEC for 18 h. B) Concentration of IL‐1*β* and IL‐23 in bladder tissues from naïve and UPEC‐infected mice was detected by ELISA (n = 3–4 mice). C) Immunohistochemical analysis of IL‐1*β* in the naïve and UPEC‐infected bladder was shown. Scale bar, 100 (top) and 25µm (bottom). D) 8 weeks‐old WT female mice were injected intravenously with either anti‐mIL‐1*β* blocking antibody (B122, 50 µg per mouse each time) or Armenian hamster IgG control at ‐10 and 0 h before infection. These mice were transurethrally infected with 1×10^8^ CFU of UPEC. At 18 h post‐infection, the bladder tissues were isolated and digested, and cells were treated with Brefeldin A (5 µg mL^−1^) for 3 h. IL‐22 and IL‐17A expression in the ROR*γ*t^+^ ILC3 were analyzed by intracellular cytokine staining. Representative plots, percentages, and absolute numbers of these cytokines‐producing cells are shown (n = 5 mice). (E–G) 8 weeks‐old WT and *Myd88* ^−/‐^ female mice were transurethrally infected with 1×10^8^ CFU of UPEC. The bladder tissues were isolated at 18 h post‐treatment. E) Concentration of IL‐1*β* in bladder tissue was detected by ELISA (n = 5 mice). F) The bladder tissues were digested and cells were treated with Brefeldin A (5 µg mL^−1^) for 3 h. IL‐22 and IL‐17A expression in the ROR*γ*t^+^ ILC3 were analyzed by intracellular cytokine staining. Representative plots, percentages, and absolute numbers of these cytokines‐producing cells are shown (n = 5 mice). G) The UPEC burdens in the whole bladder tissue are shown (n = 5 mice). Data are representative of two independent experiments. Error bars represent the mean ± SEM. Two‐way ANOVA followed by Bonferroni test (A, B, D, F), One‐way ANOVA test (E), and unpaired two‐tails student's *t*‐test (G) were used for statistical analysis, *p<0.05, **p<0.01, ***p<0.001; ns, no significant difference.

We have shown that ILC3s rapidly respond to UPEC infection and produce multiple effector cytokines. However, it is still unknown which pro‐inflammatory signals drive the activation of bladder‐resident ILC3s. We first tested the effect of IL‐1*β* and IL‐23, which are well‐known upstream stimulators of ILC3s. Total leukocytes isolated from bladder tissues were treated with IL‐1*β* and IL‐23 in vitro, and the cytokine production by ILC3s was examined with flow cytometry. Both IL‐1*β* and IL‐23 promoted the production of IL‐17A and IL‐22 in bladder ILC3s in vitro, similar to results in ILC3s from the small intestine (**Figure** **6**A). Interestingly, treatment with either IL‐1*β* or PMA/ionomycin induced a dramatic increase in the production of IL‐17, but not IL‐22, in bladder ILC3s (Figure 6A). This indicated that bladder ILC3s may primarily produce IL‐17 but not IL‐22 in situ. Furthermore, after UPEC infection, IL‐1*β* (but not IL‐23) was significantly up‐regulated at both the mRNA (Figure 3H) and protein levels (Figure 6B), suggesting that IL‐1*β* may be the key inducer of ILC3 activation in the UPEC‐infected bladder. Immunohistochemical staining of IL‐1*β* in the naïve and UPEC‐infected bladder further showed that bladder epithelial cells may be the dominant producer of IL‐1*β* post‐infection (Figure 6C). Moreover, WT mice were treated with IL‐1*β*‐blocking antibody then infected with UPEC. IL‐1*β* neutralization significantly reduced IL‐17A and IL‐22 expression in bladder ILC3s during UTI (Figure 6D), demonstrating the role of IL‐1*β* in activating ILC3s in vivo. MyD88‐dependent signaling is known to be required for pathogen recognition and triggering subsequent inflammation, including IL‐1*β* production.^[^ ^51^, ^52^ ^]^ Consistent with the IL‐1*β* blockade, *Myd88* ^−/−^ mice exhibited diminished IL‐1*β* expression in bladder tissues (Figure 6E), with almost no IL‐17A or IL‐22 production by bladder ILC3s post‐UPEC infection (Figure 6F) and uncontrolled UPEC burden during UTI (Figure 6G). Together, these data suggest that the activation of bladder‐resident ILC3s in response to the UPEC infection relies on the MyD88‐IL‐1*β* pathway.

### 2.7 Bladder‐Resident ILC3s Control Early UPEC Infection Through IL‐17A‐Mediated Neutrophil Recruitment

**Figure 7:**
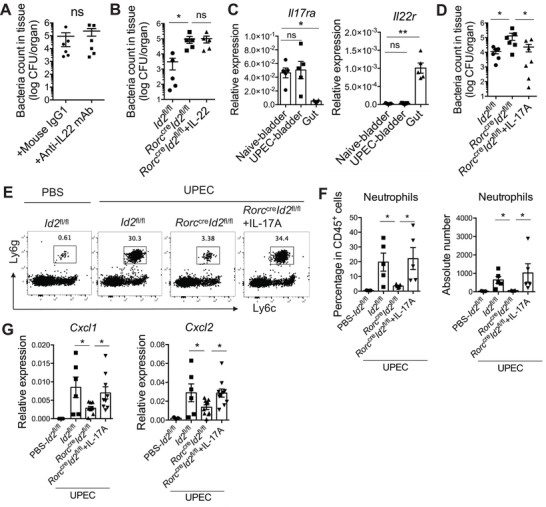
ILC3‐derived IL‐17A, but not IL‐22, could promote the UPEC elimination through recruiting the neutrophils. A) 8 weeks‐old WT female mice were intraperitoneally injected with anti‐IL‐22 mAb or mouse IgG1 (200µg per mouse each time) before UPEC infection. The UPEC burdens in the whole bladder tissue at 18 h after infection are shown (n = 6 mice). B) 8 weeks‐old *Rorc* ^cre^ *Id2* ^fl/fl^ female mice were intravenously injected with IL‐22‐Fc (500ng), and control *Id2* ^fl/fl^ and *Rorc* ^cre^ *Id2* ^fl/fl^ mice were injected with PBS, then infected with UPEC. The UPEC burdens in the whole bladder at 18 h after infection are shown (n = 5 mice). C) The mRNA expression of *Il22r* and *Il17ra* in the naive bladder, UPEC‐infected bladder, and gut are shown (n = 5 mice). D) 8 weeks‐old *Rorc* ^cre^ *Id2* ^fl/fl^ female mice were intravenously injected with recombinant IL‐17A (500ng), and control *Id2* ^fl/fl^ and *Rorc* ^cre^ *Id2* ^fl/fl^ mice were injected with PBS, then infected them by UPEC. The UPEC burdens in the whole bladder tissue at 18 h after infection are shown (n = 6–7 mice). 8 weeks‐old *Id2* ^fl/fl^, *Rorc* ^cre^ *Id2* ^fl/fl^, and IL‐17A‐injected *Rorc* ^cre^ *Id2* ^fl/fl^ female mice were infected by UPEC and compared with uninfected *Id2* ^fl/fl^ mice. The bladders at 4h post‐treatment were isolated and analyzed by flow cytometry and Real‐time qPCR. E) Representative plots show the gated neutrophils (CD45^+^Ly6c^med^Ly6g^+^ cells). F) The percentages and absolute counts of neutrophils in the whole bladder are shown (n = 5 mice). G) The mRNA expression of the indicated chemokines is shown (n = 6–10 mice). Data are representative of three independent experiments. Error bars represent the mean ± SEM. Unpaired two‐tails student's *t*‐test (A), Kruskal–Wallis test (B, C), and One‐way ANOVA test (D, F, G) were used for statistical analysis, *p<0.05, **p<0.01; ns, no significant difference.

Next, we explored how ILC3s regulated early protection against UPEC infection. IL‐22, mainly produced by ILC3s, is crucial for host defenses against various pathogens.^[^ ^4^ ^]^ To test whether IL‐22 mediated the protective role of ILC3s post‐UPEC infection, WT mice were treated with anti‐IL‐22 blocking or control antibodies. The IL‐22 blockade did not change the pathogen burden in the bladder (**Figure** **7**A), but down‐regulated the expression of antimicrobial peptides (*Reg3b* and *Reg3g*) in the gut (Figure S5A, Supporting Information), suggesting that IL‐22 is not necessary for protection against UPEC. Furthermore, *Rorc* ^cre^ *Id2* ^fl/fl^ mice were administered with IL‐22‐Fc and infected with UPEC. Although IL‐22 supplementation effectively up‐regulated the expression of *Reg3b* and *Reg3g* in the gut (Figure S5B, Supporting Information), it did not rescue ILC3‐deficient *Rorc* ^cre^ *Id2* ^fl/fl^ mice from UPEC infection (Figure 7B). This suggests that the protective role of ILC3s in UTI is not through IL‐22 signaling.

IL‐17A is another important effector cytokine of ILC3s, and protects the host from various infections including UPEC.^[^ ^33^, ^36^, ^53^, ^54^ ^]^ Interestingly, the IL‐17 receptor was highly expressed in the bladder, whereas the expression of the IL‐22 receptor was primarily enriched in the gut tissue (Figure 7C). Thus, we tested whether IL‐17A could rescue *Rorc* ^cre^ *Id2* ^fl/fl^ mice from UPEC infection. Unlike IL‐22, IL‐17A treatment significantly reduced the UPEC burden in the bladder (Figure 7D). It has been reported that IL‐17A promotes the production of chemokines CXCL1 and CXCL2 by gut epithelial cells, then recruits neutrophils for defense against gut bacterial infection.^[^ ^55^ ^]^ Compared with control mice, ILC3‐deficient *Rorc* ^cre^ *Id2* ^fl/fl^ mice also displayed reduced neutrophil accumulation (Figure 7E,F), accompanied by reduced expression of CXCL1 and CXCL2 in the bladder after UPEC infection (Figure 7G). However, IL‐17A administration successfully rescued chemokine expression and neutrophil recruitment (Figure 7E–G). Collectively, these data indicate that bladder‐resident ILC3s can control early UPEC infection through IL‐17A‐mediated neutrophil recruitment.

## 3 Discussion

ILCs are mainly distributed in the barrier tissues, where they acquire phenotypes and perform functions that are uniquely shaped by the tissue‐specific microenvironment.^[^ ^15^ ^]^ Here, we explored the signatures of distinct ILC subsets in the bladder. NK cells are the dominant ILC population in the bladder, whereas ILC1 is found at extremely low levels in the urinary tract. ILC2s respond differently to IL‐25 and IL‐33 via the corresponding receptors (IL‐25R and ST2, respectively) in various organs. Gut ILC2s are mainly the IL‐25‐responsive “inflammatory” ILC2s,^[^ ^56^ ^]^ whereas ILC2s in the lung and adipose tissues have an IL‐33‐responsive “natural” state.^[^ ^57^, ^58^ ^]^ Our results show that bladder ILC2s express high levels of ST2 but low levels of IL‐25R, suggesting a potential role of IL‐33 in the activation of bladder ILC2s. In adult mice, ILC3s can be divided into three subsets: CCR6^+^ LTi‐like cells and NCR^+^ or NCR^−^ ILC3s; the majority of ILC3s in the urinary tract were CCR6^+^ LTi‐like cells. Our previous reports showed that LTi‐like cells exhibit heterogeneity in different tissues and mainly interact with T cells through MHC‐II and PD‐L1 in mLN, while producing effector cytokines in the gut. The transcriptional regulator ID2 differentially regulates the maintenance of LTi‐like cells in mLNs and the gut. LTi‐like cells are significantly reduced in the gut of *Rorc* ^cre^ *Id2* ^fl/fl^ mice, but increased in mLN.^[^ ^3^, ^16^ ^]^ Here, our data show that bladder‐resident LTi‐like cells produce IL‐17 to protect the host from UPEC infection, express low levels of MHC‐II, and are almost completely eliminated by ID2 deficiency, suggesting that bladder‐resident ILC3s are similar to LTi‐like cells in the gut. How the bladder microenvironment imprints ILC3s and whether bladder‐resident ILC3s regulate adaptive immunity require further study.

The urinary tract has long been considered sterile, but recent reports have shown that there is a normal bladder microbiome.^[^ ^59^, ^60^ ^]^ Commensal microbiota influence ILC development, maintenance, and function.^[^ ^41^ ^]^ Previous reports show that gut ILC2s are more abundant in the germ‐free adult mice than SPF mice,^[^ ^61^, ^62^ ^]^ and our findings demonstrate a comparable change in bladder ILC2s in germ‐free mice. However, the effect of the microbiota on ILC3s is controversial. Some reports show that commensal microflora contributes to the stabilization of IL‐22‐producing NKp46^+^ ILC3s but not LTi‐like cells;^[^ ^44^, ^63^, ^64^ ^]^ another study showed that microbiota did not affect the development of ILC3s, but suppressed the production of IL‐22.^[^ ^65^ ^]^ Our studies found no change in the total number of bladder‐resident ILC3s between germ‐free and antibiotic‐treated mice. Thus, whether the microbiota influences the function of bladder‐resident ILCs requires further investigation.

ILCs play important roles in the immune defense and maintenance of the microecological balance in various barrier tissues.^[^ ^12^ ^]^ UTI is one of the most common bacterial infections worldwide and has a high recurrence, making it urgent to understand bladder‐mucosal immunity and develop new and efficacious therapies.^[^ ^25^ ^]^ Here, we found that UTI drives a rapid response in bladder ILCs, especially ILC3s. ILC‐deficient mice were more sensitive to UTI in the absence of IFN‐*γ*, IL‐17A, and IL‐22 production. Furthermore, ILC3‐deficient mice had an increased UPEC burden in the bladder during early UTI stages, suggesting a protective role of bladder ILC3s during UPEC infection. A previous report demonstrated the role of NK cells in mediating host defense against UTI through secreting TNF‐*α*.^[^ ^23^ ^]^ Our data also showed an increased NK cell response to UPEC infection. Clinically‐related studies have shown that overexpression of IL‐13 and ILC2s in the urine are associated with bladder cancer.^[^ ^21^, ^22^ ^]^ Recently, a highly polarized Th2 response to UTI has been reported to promote bladder epithelial regeneration.^[^ ^66^ ^]^ Our data show that the type 2 immune response can be induced by UPEC infection. However, this type 2 immune response may not be due to the activation of bladder‐resident ILC2s, and ILC2 deficiency does not affect early control of UPEC in the bladder. It would be worthwhile to further determine the source and role of type 2 immunity at early time points in UPEC infection, and to establish whether ILC2s are required for bladder epithelial repair or inflammation control at later infection stages.

It has been shown that IL‐17A derived from bladder‐resident *γδ*T cells can protect the host against UTI through recruiting neutrophils,^[^ ^33^ ^]^ which quickly eliminate pathogens and are also essential for controlling UPEC infection.^[^ ^32^, ^34^, ^67^ ^]^ Here, we describe ILC3s as another key source of IL‐17A. ILC3s protected the host from UPEC infection through IL‐17A‐dependent neutrophil recruitment. IL‐22 was seemingly dispensable in the early elimination of UPEC, but whether IL‐22 is involved in bladder tissue repair remains unknown. Additionally, UPEC typically establishes reservoirs in the intestine before transferring to the urinary tract and causing UTI.^[^ ^68^ ^]^ Thus, inhibition of UPEC intestinal colonization can also reduce the incidence of UTI.^[^ ^69^ ^]^ Gut ILCs, especially ILC3s, have been shown to have an important role in limiting pathogen colonization in the gut.^[^ ^13^ ^]^ Whether gut ILC3s can also control UTI through limiting early UPEC intestinal colonization requires further study.

## 4 Conclusion

In this study, we identified the unique properties of bladder‐resident ILC subsets in terms of their distribution, development, and functions. We also verified a protective role of bladder‐resident ILC3s in controlling UPEC infection, which may promote the emergence of new therapeutic strategies for UTI treatment.

## 5 Experimental Section

### Mice

C57BL/6 mice were purchased from Beijing Vital River Laboratory Animal Technology Co., Ltd. *Rag1* ^−/−^, *Rag2* ^−/−^ *Il2rg* ^−/−^, and *Rorc* ^GFP/+^ mice^[^ ^5^ ^]^ were purchased from the Jackson Laboratory. *Rorc* ^cre^ mice^[^ ^50^ ^]^ were kindly provided by Dr. Dan R. Littman (New York University, NY). *Id2* ^fl/fl^ mice^[^ ^70^ ^]^ were kindly provided by Dr. Anna Lasorella and Dr. Antonio Lavarone (Columbia University, NY). *Il5^tdtomato‐^* ^cre^ mice were kindly provided by Dr. Ju Qiu (Shanghai Institute of Nutrition and Health, Chinese Academy of Sciences). *Rorc* ^cre^ *Id2* ^fl/fl^ and *Il5^tdtomato‐^* ^cre^ *Id2* ^fl/fl^ mice were generated by crossing *Rorc* ^cre^ and *Il5^tdtomato‐^* ^cre^ mice with *Id2* ^fl/fl^ mice. Crossing *Rorc* ^cre^ *Id2* ^fl/fl^ mice and *Id2* ^fl/fl^ mice with *Rorc* ^GFP/+^ mice generated *Rorc* ^cre^ *Id2* ^fl/fl^ *Rorc* ^GFP/+^ mice and *Id2* ^fl/fl^ *Rorc* ^GFP/+^ mice. Crossing *Rorc* ^cre^ *Id2* ^fl/fl^ mice and *Id2* ^fl/fl^ mice with *Rag1* ^−/−^ mice generated *Rag1* ^−/−^ *Rorc* ^cre^ *Id2* ^fl/fl^ mice and *Rag1* ^−/−^ *Id2* ^fl/fl^ mice. *Myd88* ^−/‐^ mice were kindly provided by Dr. Zhihua Liu (Tsinghua University, Beijing) and Xiaoyu Hu (Tsinghua University, Beijing). All mice were on C57BL/6 background and maintained under specific pathogen‐free conditions at the Tsinghua University. Germ‐free mice were maintained in the gnotobiotic facility at Tsinghua University. All animal studies were approved by the Animal Care and Use Committee of the Tsinghua University (Approval number: IACUC‐15‐GXH1).

### Antibiotics‐Treated Experiments

8 weeks‐old C57BL/6 mice were treated with antibiotics, including ampicillin (1 g L^−1^), neomycin (1 g L^−1^), metronidazole (1 g L^−1^), and vancomycin (0.5 g L^−1^), in drinking water for 3 weeks.

### UPEC‐Induced UTI and Determination of Bacterial Burden

8–10 weeks‐old female mice were transurethrally catheterized for the inoculation of UPEC under the anesthesia of avertin. UPEC strain was a clinical isolate *E. coli* CFT073, and 1×10^8^ CFU in sterile PBS (50 µL) was used for all infections.^[^ ^71^ ^]^ Bladder tissues were washed by sterile PBS, homogenized in TritonX‐100 (0.1%), serially diluted, and spread on MaCONKEY plates. Bacteria colonies on plates were counted after overnight incubation at 37 °C.

### Recombinant IL‐17A, IL‐22‐Fc, Anti‐IL‐22, and Anti‐IL‐1*β* Antibody Treatment

The recombinant mouse IL‐17A was purchased from Biolegend company. The IL‐22‐Fc and the monoclonal antibody against murine IL‐22 (8E11.9) used in this study have been previously described.^[^ ^3^, ^72^ ^]^ The monoclonal antibody against mouse/rat IL‐1*β* (B122, BE0246) was purchased from BioXcell. Recombinant mouse IL‐17A (500 ng) or IL‐22‐Fc (500 ng) was injected intravenously into *Rorc* ^cre^ *Id2* ^fl/fl^ mice at 1h before the UPEC infection, meanwhile the control mice were injected by PBS. WT female mice were injected intraperitoneally with anti‐IL‐22 mAb or mouse IgG1 (200 µg per mouse each time) as an isotype control at day ‐4, ‐2, and 0 post‐infection of UPEC. WT female mice were injected intravenously with either anti‐mIL‐1*β* blocking antibody or Armenian hamster IgG control (50µg per mouse each time) at ‐10 and 0 h before infection.

### Isolation of Bladder, Kidney Cells, and Intestinal LPLs

Bladder or kidney tissues were cut into 1mm pieces, washed by sterile PBS, and digested in RPMI 1640 medium (Invitrogen) containing DNase I (0.05%, Sigma) and Liberase TL (0.1 mg mL^−1^, Roche) at 37 °C (30 min for bladder and 20 min for kidney). The digested tissues were homogenized by the gentleMACS Dissociator (Miltenyi Biotec) and passed through a cell strainer (70 µm). Single‐cell suspensions of bladder were directly collected, whereas single‐cell suspensions of kidney need to be harvested from the interphase of an 80% and 40% Percoll (GE) gradient after a spin at 2000 rpm for 20 min at room temperature, then for further analysis by flow cytometry. The isolation of intestinal LPLs was done as previously described.^[^ ^73^ ^]^

### In Vitro Stimulation of ILCs in Bladder or Small Intestine

Total leukocytes isolated from bladder tissue or the lamina propria of the small intestine were stimulated in vitro with IL‐1*β* (10 ng mL^−1^, PeproTech), IL‐23 (10 ng mL^−1^, PeproTech) or PMA (50 ng mL^−1^, Sigma), and ionomycin (750 ng mL^−1^, Calbiochem) for 4 h. Brefeldin A (5 µg mL^−1^, Biolegend) was added 3 h before cells were harvested for analysis. The cytokine production by ILCs was further detected with flow cytometry.

### Flow Cytometry

For nuclear staining, cells were fixed and permeabilized with a Transcription Factor Staining Buffer Set (eBioscience). For cytokine detection, cells were treated in vitro with different stimulators for 4 h and Brefeldin A (5 µg mL^−1^, Biolegend) was added 3 h before cells were harvested for antibody staining. IC Fixation Buffer and Permeabilization Buffer (eBioscience) were used for intracellular cytokine staining. Flow cytometry was performed on BD LSRFortessa (BD Biosciences) instruments and analyzed with FlowJo software (Tree Star). The fixable viability dyes eFluor780 or eFluor506 (Thermo Fisher) were used to exclude dead cells.

### List of Antibodies used for Flow Cytometry

See Table S1, Supporting Information.

### ELISA

Concentrations of mIL‐1*β* (Invitrogen, 88‐7013‐22) and mIL‐23 (eBioscience, 88‐7230‐22) were measured by ELISA kit according to the manufacturer's recommendations.

### Histological Staining

Bladder samples were fixed in PFA (4%), embedded in paraffin, cut for 5‐µm sections, and stained with anti‐mIL‐1*β* antibody (RM1009, abcam, ab283818).

### Quantitative Real‐Time RT‐PCR

RNA from bladder tissues was extracted with a Total RNA isolation kit (Axygen). RNA (1 µg) was used for the cDNA synthesis with Reverse Transcription Kit (Thermo). Real‐time qPCR was performed with qPCR SYBR Green Master Mix (YEASEN) and different primer pairs on StepOne Plus (Applied Biosystems). Target gene expression was normalized to *β*‐actin and determined using the 2^−ΔΔ*CT*^ method.

### List of Primer Sequences used for Real‐Time RT‐PCR

See Table S2, Supporting Information.

### Statistical Analysis

The sample sizes (n), probability (p) value, and the specific statistical test for each experiment were indicated in the figure legends. For the comparison between two groups, unpaired two‐tailed Student's *t*‐test was used. For multiple comparisons among different groups, one‐way ANOVA or two‐way ANOVA followed by Bonferroni test was used. In all cases, Continuous variables were expressed as mean ± SEM; *p*<0.05 was considered significant (*p<0.05, **p<0.01, ***p<0.001; ns, no significant difference). Statistical analysis was carried out using GraphPad Prism 6.0 program. No data were excluded from statistical analysis. Data presented were representative of at least two independent experiments.

## Conflict of Interest

The authors declare no conflict of interest.

## Author Contributions

G.X. is the senior and corresponding author. G.X. conceived and designed the study. G.X. and H.J. prepared the manuscript. H.J. performed all experiments and assisted in data analysis. F.L., H.J., Z.J., Z.X., W.W., L.Y., and S.B. participated in some experiments. Q.J., H.X., and L.Z. provided critical materials.

## Supporting information

Supporting Information

## Data Availability

The data that support the findings of this study are available from the corresponding author upon reasonable request.
