# Supplementary material for: Group 3 Innate Lymphoid Cells Protect the Host from the Uropathogenic Escherichia coli Infection in the Bladder
Source: Adv Sci (Weinh). 2022 Jan 12;9(6):2103303. doi: 10.1002/advs.202103303 (PMC8867143; doi:10.1002/advs.202103303)
Supplement: Supplementary file 1 — Supporting Information [file ADVS-9-2103303-s001.pdf]

ADVANCED SCIENCE
Open Access

Supporting Information

for *Adv. Sci.*, DOI: 10.1002/advs.202103303

Group 3 Innate Lymphoid Cells Protects the Host from the Uropathogenic *Escherichia coli* Infection in the Bladder

Jiaoyan Huang, Liuhui Fu, Jida Huang, Jie Zhao, Xin Zhang, Wenyan Wang, Yeyang Liu, Bowen Sun, Ju Qiu, Xiaoyu Hu, Zhihua Liu, and *Xiaohuan Guo**

Supporting Information

Group 3 Innate Lymphoid Cells Protect the Host from the Uropathogenic *Escherichia coli* Infection in the Bladder

Jiaoyan Huang, Liuhui Fu, Jida Huang, Jie Zhao, Xin Zhang, Wenyan Wang, Yeyang Liu, Bowen Sun, Ju Qiu, Xiaoyu Hu, Zhihua Liu, Xiaohuan Guo[*]

The Supplementary Materials include:

Figure S1. Distribution of ILC subsets in the kidney.

Figure S2. Both commensal and sex did not influence the maintenance of kidney-resident ILCs.

Figure S3. Bladder ILC2 is not required for the early elimination of UPEC.

Figure S4. RAG-deficient mice exhibit increased ILC3 response in the bladder.

Figure S5. IL-22 blockade or supplementation effectively regulated the expression of antimicrobial peptides in the gut.

Table S1. Antibodies used for flow cytometry.

Table S2. Primer sequences used for real-time RT-PCR.

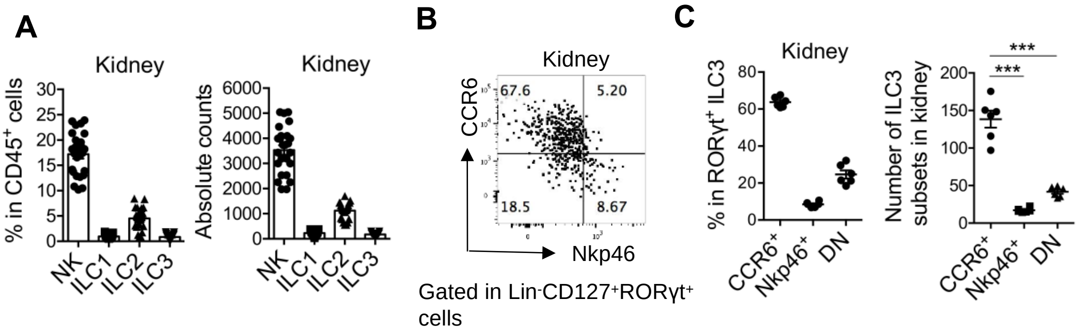

Figure S1. Distribution of ILC subsets in the kidney.

A) The percentages in $CD45^+$ cells and absolute numbers of different ILC subsets from the kidney of WT mice are shown (n=25 mice). Data shown are representative of five independent experiments. B) Representative plot shows CCR6 and NKp46 expression on the $ROR\gamma t^+$ ILC3 in the kidney of WT mice. C) The percentages in total $ROR\gamma t^+$ ILC3 and absolute numbers of ILC3 subsets ($CCR6^+$, $NKp46^+$ and $CCR6^-NKp46^-$ cells) from the kidney of WT mice are shown (n=6 mice). Data are pooled from two independent experiments. Data are shown as the mean ± SEM. One-way ANOVA test (C) was used for statistical analysis, ***p<0.001. Each dot represents one individual mouse.

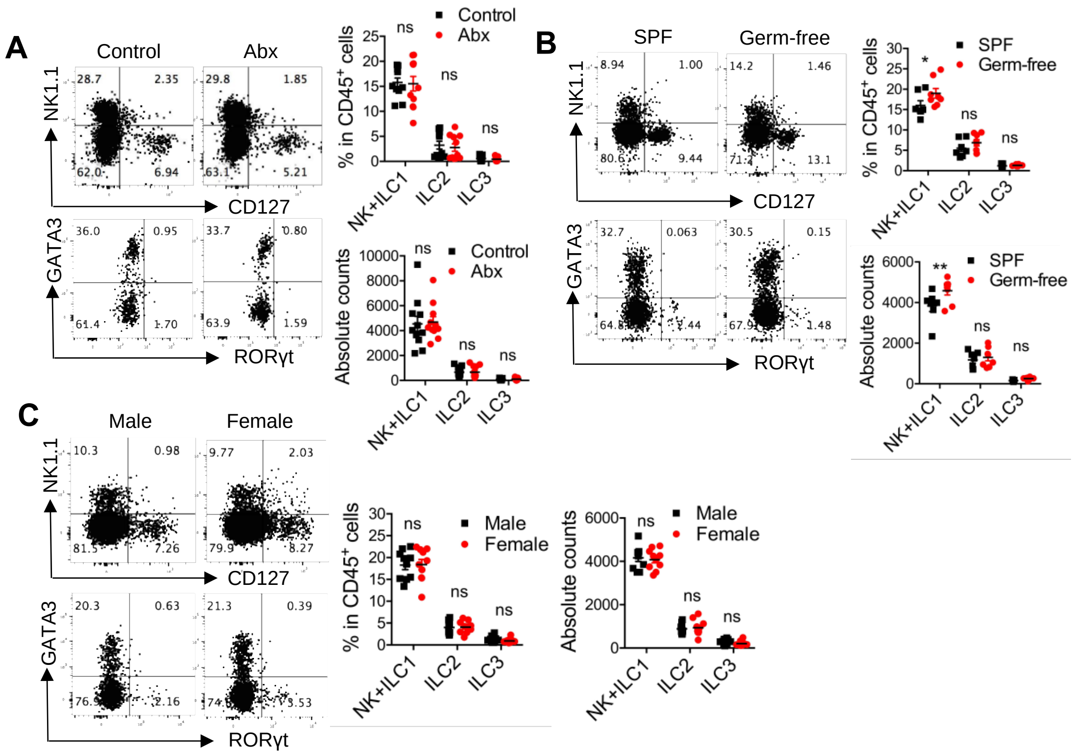

Figure S2. Both commensal and sex did not influence the maintenance of kidney-resident ILCs.

A) Representative plots, percentages and absolute numbers of different ILC subsets in the kidney of the antibiotics-treated (Abx) mice and the control C57BL/6 mice are shown (n=11 mice). B) Representative plots, percentages and absolute numbers of different ILC subsets in the kidney of germ-free mice and SPF C57BL/6 mice at the same age are shown (n=8 mice). C) Representative plots, percentages and absolute numbers of different ILC subsets in the kidney of male and female C57BL/6 mice at the same age are shown (n=10 mice). Mice in these experiments are adult. Data are pooled from two independent experiments, and shown as mean ± SEM. Two-way ANOVA followed by Bonferroni test was conducted for statistical analysis. *$p<0.05$, **$p<0.01$; ns, no significant difference.

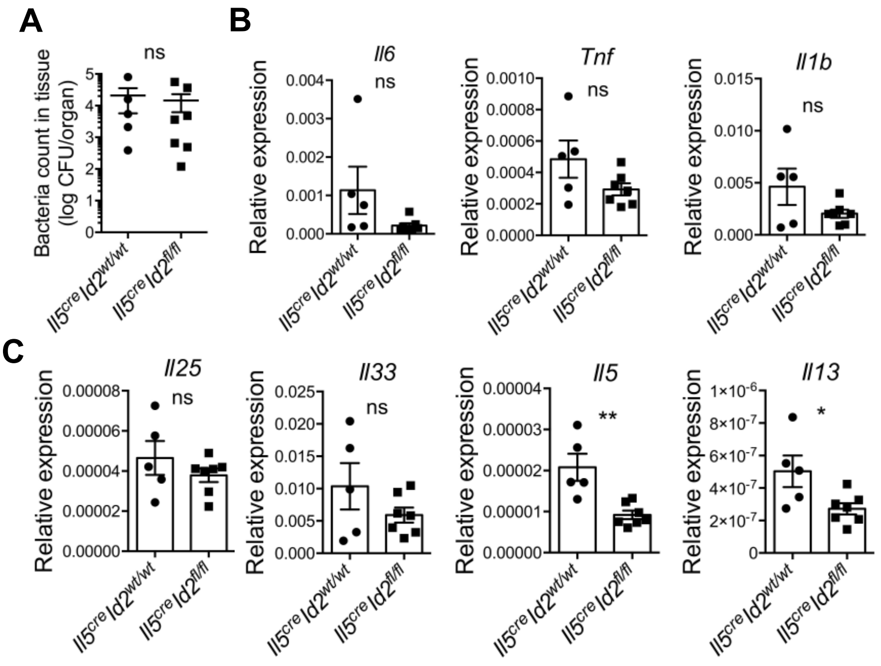

Figure S3. Bladder ILC2 is not required for the early elimination of UPEC.

8 weeks-old $Il5^{\text{tdtomato-cre}}Id2^{\text{wt/wt}}$ and $Il5^{\text{tdtomato-cre}}Id2^{\text{fl/fl}}$ female mice were transurethrally infected with $1*10^8$ CFU of UPEC. The bladder tissues at 18h post treatment was isolated. A) The UPEC burdens in the whole bladder tissue are shown (n=5-7 mice). B) The mRNA expression of the indicated pro-inflammation cytokines in the infected bladder tissues are shown (n=5-7 mice). C) The mRNA expression of the indicated type 2 cytokines in the infected bladder tissues are shown (n=5-7 mice). Data are representative of three independent experiments. Error bars represent the mean ± SEM. Unpaired two-tails student's *t*-test was used for statistical analysis, *p<0.05, **p<0.01; ns, no significant difference.

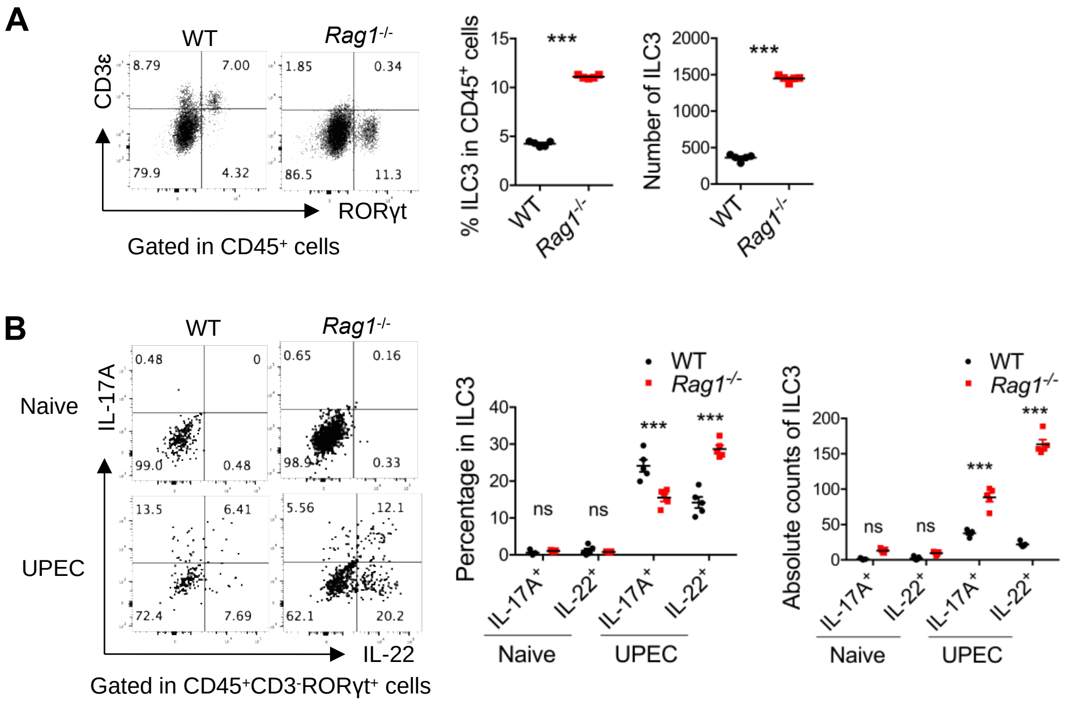

Figure S4. RAG-deficient mice exhibit increased ILC3 response in the bladder.

A) Representative plots, percentages and absolute numbers of ILC3 (CD45^{+}CD3ε^{-}RORγt^{+} cells) in the bladder of naïve WT and *Rag1*$^{-/-}$ mice are shown (n=5 mice). B) 8 weeks-old WT and *Rag1*$^{-/-}$ female mice were transurethrally infected with $1*10^{8}$ CFU of UPEC for 18 h. The naïve and UPEC-infected bladder tissues were digested and cells were treated with Brefeldin A (0.5µg/ml) for 3 h. IL-22 and IL-17A expression in the RORγt^{+} ILC3 were analyzed by intracellular cytokine staining. Representative plots, percentages and absolute numbers of these cytokines-producing cells are shown (n=5 mice). Data are representative of two independent experiments, and shown as mean ± SEM. Unpaired two-tails student's *t*-test (A) and Two-way ANOVA followed by Bonferroni test (B) was conducted for statistical analysis. ***p<0.001; ns, no significant difference.

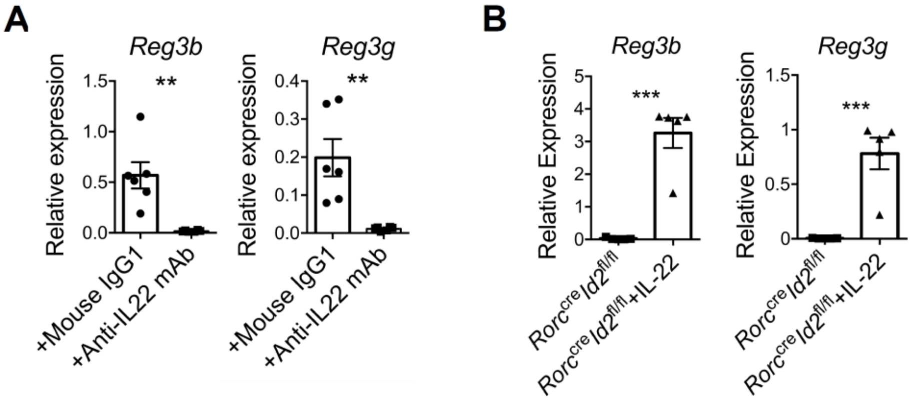

Figure S5. IL-22 blockade or supplementation effectively regulated the expression of antimicrobial peptides in the gut.

A) Related to Figure 7A, 8 weeks-old WT female mice were intraperitoneally injected with anti-IL-22 mAb or mouse IgG1 (200μg per mouse each time). The mRNA expression of *Reg3b* and *Reg3g* in the gut of these mice was detected (n=6 mice). B) Related to Figure 7B, 8 weeks-old *Rorc*^{cre}*Id2*$^{fl/fl}$ female mice were intravenously injected with IL-22-Fc (500ng) or PBS. The mRNA expression of *Reg3b* and *Reg3g* in the gut of these mice was detected (n=5 mice). Data are representative of two independent experiments. Error bars represent the mean ± SEM. Unpaired two-tails student's *t*-test (A) and One-way ANOVA test (B) was used for statistical analysis, **p<0.01, ***p<0.001.

Table S1. Antibodies used for flow cytometry.

Antibodies	Source	Identifier
FITC anti-mouse CD3ε (Clone 145-2C11)	Biolegend	Cat# 100306; RRID: AB_312671
APC anti-mouse CD3ε (Clone 500A2)	Biolegend	Cat# 152306; RRID: AB_2632669
Biotin anti-mouse CD3ε (Clone 145-2C11)	Biolegend	Cat# 100304; RRID: AB_312669
Brilliant Violet 605™ anti-mouse CD4 (Clone GK1.5)	Biolegend	Cat# 100451; RRID: AB_2564591
Biotin anti-mouse CD4 (Clone GK1.5)	Biolegend	Cat# 100404; RRID: AB_312689
Alexa Fluor® 700 anti-mouse CD8a (Clone 53-6.7)	Biolegend	Cat# 100730; RRID: AB_493703
Biotin anti-mouse CD8a (Clone 53-6.7)	Biolegend	Cat# 100704; RRID: AB_312743
Alexa Fluor® 700 anti-mouse CD45 (Clone 30-F11)	Biolegend	Cat# 103128; RRID: AB_493715
Alexa Fluor® 700 anti-mouse CD45.2 (Clone 104)	Thermo Fisher Scientific	Cat# 56-0454-82; RRID: AB_657752
FITC anti-mouse CD90.2 (Clone 30-H12)	Biolegend	Cat# 105306; RRID: AB_313177
Brilliant Violet 510™ anti-mouse CD90.2 (Clone 53-2.1)	Biolegend	Cat# 140319; RRID: AB_2561395
PE anti-mouse CD127 (Clone SB/199)	Biolegend	Cat# 121112; RRID: AB_493509
Brilliant Violet 421™ anti-mouse CD127 (Clone A7R34)	Biolegend	Cat# 135027; RRID: AB_2563103
Brilliant Violet 605™ anti-mouse Ly-6G (Clone 1A8)	Biolegend	Cat# 127639; RRID: AB_2565880
Alexa Fluor 700 anti-mouse Ly-6c Antibody (Clone HK1.4)	Biolegend	Cat# 128024; RRID: AB_10643270
Biotin anti-mouse Ly-6c/Ly-6c (Gr-1) (Clone RB6-8C5)	Biolegend	Cat# 108404; RRID: AB_313369
Biotin anti-mouse/human CD45R/B220 (Clone RA3-6B2)	Biolegend	Cat# 103204; RRID: AB_312989
PerCP/Cyanine5.5 anti-mouse NK-1.1 (Clone PK136)	Biolegend	Cat# 108728; RRID: AB_2132705
Biotin anti-mouse NK-1.1 (Clone PK136)	Biolegend	Cat# 108704; RRID: AB_313391
APC anti-mouse NKP46 Antibody (Clone 29A1.4)	Biolegend	Cat# 137608; RRID: AB_10612758

Biotin anti-mouse TCRβ (Clone H57-597)	Thermo Fisher Scientific	Cat# 13-5961-82; RRID: AB_466819
Biotin anti-mouse TCRγδ (Clone GL3)	Thermo Fisher Scientific	Cat# 13-5711-82; RRID: AB_466668
Biotin anti-mouse TER-119/Erythroid Cells (TER-119)	Biolegend	Cat# 116204; RRID: AB_313705
APC anti-mouse IL-33Rα (IL1RL1, ST2) (Clone DIH9)	Biolegend	Cat# 145306; RRID: AB_2561917
APC anti-mouse CCR6 Antibody (Clone 29-2L17)	Biolegend	Cat# 129813; RRID: AB_1877148
Alexa Fluor 488 anti-mouse CCR7 Antibody (Clone 4B12)	Biolegend	Cat# 120110; RRID: AB_492841
PE anti-mouse CXCR6 Antibody (Clone K041E5)	Biolegend	Cat# 151104; RRID: AB_2566546
FITC anti-mouse CD25 Antibody (Clone 3C7)	Biolegend	Cat# 101908; RRID: AB_961212
eFluor 660 anti-mouse IL-25R Antibody (Clone MUNC33)	eBioscience	Cat# 50-7361-82; RRID: AB_2574289
APC/Cy7 anti-mouse Sca-1 Antibody (Clone D7)	Biolegend	Cat# 108126; RRID: AB_10645327
PerCP/Cy5.5 anti-mouse c-Kit Antibody (Clone 2B8)	Biolegend	Cat# 105824; RRID: AB_2131597
Pacific Blue anti-mouse I-A/I-E Antibody (Clone M5/114.15.2)	Biolegend	Cat# 107620; RRID: AB_493527
Biotin anti-mouse RANK Antibody (Clone R12-31)	Biolegend	Cat# 119803; RRID: AB_2205355
PerCP/Cy5.5 anti-mouse PD1 Antibody (Clone 29F.1A12)	Biolegend	Cat# 135208; RRID: AB_2159184
Biotin anti-mouse PD-L1 Antibody (Clone 10F.9G2)	Biolegend	Cat# 124306; RRID: AB_961220
APC anti-mouse Integrin α4β7 Antibody (Clone DATK32)	Biolegend	Cat# 120608; RRID: AB_10730607
Alexa Fluor® 647 Mouse IgG2a, κ Isotype Ctrl (Clone MOPC-173)	Biolegend	Cat# 400240
Alexa Fluor® 488 Mouse IgG1, κ Isotype Ctrl (Clone MOPC-21)	Biolegend	Cat# 400134
PE anti-mouse/human GATA3 (Clone TWAJ)	Thermo Fisher Scientific	Cat# 12-9966-42; RRID: AB_1963600
eFluor 660 anti-mouse/human GATA3 (Clone TWAJ)	Thermo Fisher Scientific	Cat# 50-9966-42; RRID: AB_10596663
PerCP-eFluor 710 anti-mouse RORγt Antibody (Clone B2D)	eBioscience	Cat# 46-6981-82; RRID: AB_ 10717956

PE/Cy7 anti-mouse T-bet Antibody (Clone 4B10)	eBioscience	Cat# 25-5825-82; RRID: AB_ 11042699
Brilliant Violet 421™ anti-mouse/human IL-5 (Clone TRFK5)	Biolegend	Cat# 504311; RRID: AB_2563161
Alexa Fluor® 488 anti-mouse IL-13 (Clone eBio13A)	Thermo Fisher Scientific	Cat# 53-7133-82; RRID: AB_2016708
PE anti-mouse IL-17A (Clone TC11-18H10.1)	Biolegend	Cat# 506904; RRID: AB_315464
FITC anti-mouse TNFα Antibody (Clone MP6-XT22)	Biolegend	Cat# 506304; RRID: AB_315425
eFluor 450 anti-mouse IFNγ Antibody (Clone XMG1.2)	eBioscience	Cat# 48-7311-82; RRID: AB_ 1834366
PerCP-eFluor 710 anti-mouse IL-22 Antibody (Clone IL22JOP)	eBioscience	Cat# 46-7222-82; RRID: AB_ 2573839
PE anti-mouse GM-CSF Antibody (Clone MP1-22E9)	BD	Cat# 554406

Table S2. Primer sequences used for real-time RT-PCR.

Gene	Forward primer	Reverse primer
Actb	AAGTGTGACGTTGACATCCG	GATCCACATCTGCTGGAAGG
Il1b	GAAATGCCACCTTTTGACAGTG	TGGATGCTCTCATCAGGACAG
Il5	CCCACGGACAGTTTGATTCT	GCAATGAGACGATGAGGCTT
Il6	TAGTCCTTCCTACCCCAATTTCC	TTGGTCCTTAGCCACTCCTTC
Il12p35	CACCCTTGCCCTCCTAAACC	CACCTGGCAGGTCCAGAGA
Il13	TCTGAAGGACTCTGGCTTTG	GATGGATGCTACCAAACTGGA
Il18	GACTCTTGCGTCAACTTCAAGG	CAGGCTGTCTTTTGTCAACGA
Il17a	CTCCAGAAGGCCCTCAGACTAC	AGCTTTCCCTCCGCATTGACACAG
Il22	CATGCAGGAGGTGGTACCTT	CAGACGCAAGCATTTCTCAG
Il23p19	GGTGGCTCAGGGAAATGT	GACAGAGCAGGCAGGTACAG
Il25	ACAGGGACTTGAATCGGGTC	TGGTAAAGTGGGACGGAGTTG
Il33	CCTCCCTGAGTACATACAATGACC	GTAGTAGCACCTGGTCTTGCTCTT
Ifng	GAGCTCATTGAATGCTTGGC	GCGTCATTGAATCACACCTG
Tnfa	CCCTCACACTCAGATCATCTTCT	GCTACGACGTGGGCTACAG
Csf2	GCAATTTCACCAAACTCAAGG	CTCATTACGCAGGCACAAAAG
Cxcl1	GCCAATGAGCTGCGCTGTCAGTGC	CTTGGGGACACCTTTTAGCATCTT
Cxcl2	CCAACCACCAGGCTACAGG	GCGTCACACTCAAGCTCTG
Ccl2	GTCCCTGTCATGCTTCTGG	GCGTTAACTGCATCTGGCT
